# Hernie inguinale en Afrique subsaharienne: quelle place pour la technique de Shouldice?

**DOI:** 10.11604/pamj.2015.22.50.6803

**Published:** 2015-09-18

**Authors:** Drissa Traoré, Lasseny Diarra, Bréhima Coulibaly, Brehima Bengaly, Birama Togola, Alhassane Traoré, Hamady Traoré, Nouhoum Ongoïba, Filifing Sissoko, Abdel Karim Koumaré

**Affiliations:** 1 Faculté de Médecine et d'Odonto Stomatologie de Bamako, Mali; 2 Institut Africain de Formation en Pédagogie, Recherche et Evaluation en Sciences de la Santé (IAFPRESS) Bamako, Mali; 3 Service de Chirurgie B CHU du Point G, Mali; 4 Service de Chirurgie Générale CHU Gabriel Touré, Mali; 5 Service de Chirurgie CHU-OS, Mali

**Keywords:** Hernie inguinale, technique de Shouldice, récidives, Inguinal hernia, Shouldice technique, relapses

## Abstract

L'objectif était d’étudier l'aspect épidémio-clinique et thérapeutique de la hernie inguinale selon la technique de Shouldice. Nous avons réalisé une étude rétrospective, portant sur les patients opérés pour hernie inguinale selon la technique de Shouldice dans le service de chirurgie B du CHU du Point G, Bamako, Mali. Il a été enregistré 225patients opérés selon la technique de Shouldice. L’âge moyen était de 49 ans +/- 17,7. Il y avait 90,7% (204) hommes soit un sex-ratio de 9,7. Les cultivateurs, les ménagères et les ouvriers ont représenté 51,1% (115). Dans 75,2% (169) les patients ont consulté pour tuméfaction inguinale. En pré opératoire, la hernie était compliquée chez 82 (36,4%) patients dont 24 cas de récidive. L’étranglement herniaire a été la principale complication pré opératoire 58,5% (48/82). Les suites opératoires à un an ont été simples chez 94,2%(210) des patients; elles étaient marquées par 8 cas de récidive, 4 cas de névralgie, 2 cas d'atrophie testiculaire, 1 cas de chéloïde. La technique de Shouldice est la technique de choix pour la cure de la hernie inguinale dans les pays en voie de développement à cause du bon résultat et son coût peu onéreux par rapport aux autres techniques utilisant des dispositifs médicaux.

## Introduction

**Figure 1:**
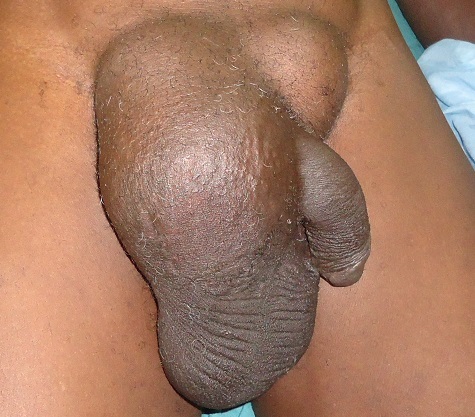
Hernie inguinale bilatérale

La hernie inguinale se définit comme une hernie qui fait issue par le canal inguinal au-dessus de l'arcade crurale (Figure 1) [1]. Près de 95% des hernies de l'aine sont des hernies inguinales qui affectent surtout le sujet masculin entre 20 et 60 ans [2]. Elles représentent 10% des interventions en chirurgie digestive. C'est une des pathologies les plus fréquentes en chirurgie générale particulièrement en Afrique où elle touche environ 4,6% de la population [3]. Le traitement des hernies inguinales est essentiellement chirurgical. Si le diagnostic des hernies inguinales est simple, les modalités de leur prise en charge restent discutées. Le traitement des hernies inguinales pose aujourd'hui la question du choix parmi plusieurs techniques chirurgicales offrant des résultats cliniques comparables mais des résultats fonctionnels et économiques différents [2]. En 1996, Simmons a conclu que *« l'intervention de Shouldice est la meilleure technique conventionnelle de cure de la hernie inguinale »* [4]. Parmi les techniques de suture ou Herniorraphie, la technique de Shouldice est considérée comme le procédé de référence en raison du taux de récidives inférieur à 1% publié par l’École de Toronto [5]. Dans notre pays voire dans les pays en voie de développement, la majorité des cures de la hernie inguinale se fait par les techniques conventionnelles. Les objectifs de cette étude étaient: d’étudier l'aspect épidémio-clinique et thérapeutique de la hernie inguinale selon la technique de Shouldice.

## Méthodes

L’étude s'est déroulée dans le service de chirurgie B du Centre Hospitalier Universitaire du Point G. Il s'agissait d'une étude rétrospective qui a porté sur 15 ans de Janvier 1995 à Décembre 2009. Ont été inclus dans notre étude, les patients opérés d′une hernie inguinale selon la technique de Shouldice dans le service chirurgie B. Etaient exclus de cette étude, les patients opérés selon la technique de Shouldice en dehors du service de chirurgie B ou opérés selon d'autres techniques. L’échantillonnage a été exhaustif durant le période d’étude.

La collecte des données a été faite à travers les fiches d'enquêtes qui ont été remplies à partir des dossiers préétablis pour chaque malade, du registre de consultation et des cahiers de compte rendu opératoire. Les patients ont été recherchés en allant à leurs domiciles ou en les téléphonant. Cette méthodologie nous a permis de revoir la majorité de nos patients. La variable dépendante était l'existence de la hernie inguinale opérée selon la technique de shouldice. Les variables indépendantes ou explicatives étaient les renseignements socio-démographiques, clinique, thérapeutique et du suivi postopératoire. La saisie a été faite sur le logiciel Excel, et l'analyse faite par le logiciel Epi info 7. Les tests statistiques utilisés ont été le Khi2 et le Fischer exact. La valeur de p inférieure à 0,05 était considérée significative.

## Résultats

Nous avons collecté 225 patients dont l’âge moyen était de 49 ans ± 17 ans avec les extrêmes de 16 et de 86 ans. La majorité de nos patients étaient de sexe masculin soit 90,7% (204) et 9,3% (21) de sexe féminin. Lesex-ratio était de 9,7. Les patients faisaient des activités physiques intenses (cultivateur, ménagère, ouvrier) dans 51,1% (115) des cas et 21,3% (48) avaient une activité intellectuelle (fonctionnaire, étudiant/élève). Les motifs de consultation ou de référence de nos patients étaient essentiellement la tuméfaction inguinale dans 75,2% (169) des cas, suivi de la tuméfaction inguinale douloureuse dans 5,8% des cas (13). On a trouvé des facteurs favorisants (effort intense, multiparité, dysurie, constipation et obésité) chez 61% (137) des patients. Dans 58,2% (131) des cas la hernie inguinale était à droite; 35,1% (79) avaient une hernie inguinale gauche et 6,7% (15) avaient une hernie inguinale bilatérale.

En pré opératoire, la hernie était compliquée chez 39,6% (89) de nos patients. Les types de complication pré opératoire étaient l’étranglement herniaire chez 53,9% (48/89) de nos patients, suivi de la récidive 34,9% (31/89) et de l'engouement herniaire 11,2% (10/89). Les patients ont été opérés sous anesthésie locale dans 48,9% (110), sous anesthésie locorégionale dans 40,4% (91) et sous anesthésie générale dans 10,7% (24) des cas. Nous avons trouvé que 85,8% (193) des patients étaient opérés par un chirurgien qualifié et 14,2% (32) par un apprenant chirurgien. En per opératoire, le sac herniaire contenait du grêle dans 14,2% (32), de l’épiploon dans 6% (13), du côlon dans 5% (11), du grêle plus le côlon dans 3,6% (8) et du grêle plus l’épiploon dans 2,2% (5) des cas.

En per opératoire, nous avons trouvé deux incidents, une lésion du côlon et une lésion du grêle. Le fil non résorbable a été utilisé chez 93% (209) des patients et le fil résorbable chez 7% (16) des patients. Le séjour post opératoire moyen a été de 5,9 jours avec des extrêmes de 1 et de 34 jours et un écart type de 3,6. Les suites opératoires immédiates (0 à 8 jours) étaient marquées par un hématome inguinal ou scrotal chez 8% (18), une douleur aiguë chez 5,3% (12) et une inflammation dans 3,6% (8) des cas.

A un mois postopératoire, on a eu un taux de morbidité de 5,8% (13), fait de 46,2%(6/13) d'abcès de la paroi, 23,1% (3/13) de retard de cicatrisation, 15,3% (2/13) de névralgie, 7,7(1/13) d'occlusion intestinale aiguë et 7,7% (1/13) de douleur testiculaire. La nature de la complication à 6 mois était la névralgie dans 46,1% (6), la chéloïde dans 23,1% (3), l'atrophie testiculaire dans 15,4% (2), la douleur testiculaire dans 7,7% (1) et le granulome sur fil dans 7,7% (1). On avait revu 100% de nos patients à un mois, 64% à un an, 45,8% à deux ans et seulement 2,3% en 14 ans de recul. A un an on a eu 3,5% (8) de récidive. A deux ans la récidive était constatée chez 2,6%(6) de nos patients. À 5 ans, post opératoire la récidive était constatée chez 1,7% (4) de nos patients revus. A 10 ans 0,4%(1). Après 10 ans on n'a trouvé aucun cas de récidive.

La récidive herniaire post opératoire n’était pas statistiquement liée à l'effort physique chez nos patients opérés (p = 0,9). Concernant le sexe et la récidive postopératoire, il n′existait pas de différence statistiquement significative entre les 2 sexes (p = 0,8). Nous n'avons pas trouvé de relation statistiquement significative entre la récidive postopératoire et la qualification du premier opérateur (p = 0,9). L’étranglement de la hernie n’était pas lié à la récidive postopératoire (p = 0,5). Il n′existait pas de différence statistiquement significative entre la récidive pré opératoire et la récidive postopératoire avec un p = 0,2. Aucun cas de décès lié au traitement de la hernie inguinale selon la technique de Shouldice n'a été enregistré.

## Discussion

Nous avons trouvé une fréquence élevée de la hernie inguinale chez les personnes relativement jeunes avec un âge moyen de 49 ans. La plus part de ces personnes était de sexe masculin soit 90,7% avec un sex-ratio de 9,7. Certains auteurs ont expliqué cette prédominance masculine, par une différence anatomique entre les deux sexes [6]. Chez l′homme le canal inguinal est traversé par le cordon qui le rend fragile. Ce qui n′est pas le cas chez la femme dont le canal inguinal ne contient que le ligament rond. Dans les pays en voie de développement cette prédominance masculine pourrait aussi s′expliquer par la difficulté qu′ont les femmes pour accéder aux soins de santé. Ce résultat pourrait être expliqué par le fait qu′en Afrique notamment au Mali, la majorité des populations vit exclusivement des travaux agricoles qui sollicitent beaucoup les muscles de la paroi abdominale. Ce qui est corroboré par l’étude de Boukinda et al [7] au Centre Hospitalier de Talangai à Brazzaville qui ont trouvé un âge moyen de 40,7 ans avec une prédominance masculine de 84,2% des cas.

Dans ce travail, 51,1% des patients faisaient des activités physiques intenses. Les différentes études menées ont monté que les travailleurs de force sont majoritairement représentés, ce qui est un argument pour étayer la théorie qui affirme que la hernie acquise est liée à l'effort physique répété qui, provoquant chaque fois une hypertension intra-abdominale, chasse les viscères mobiles vers les zones herniaires déhiscentes où ils s'extériorisent progressivement [8]. La principale complication de la hernie inguinale est l’étranglement, nous avons trouvé 21,33%. Ce taux est inférieur à celui de Mehinto et al [9] qui ont trouvé 26,4% (p = 0,003) de hernie étranglée sur 432cas. Cette différence peut être due à la taille de l’échantillon qui est plus importante dans leur étude et le mode de recrutement d’étranglement herniaire. Le risque d’étranglement d'une hernie au cours de son évolution est de 3 à 7% [10]. Dans 58,2% des cas la hernie était à droite; ce qui est semblable à ceux obtenus par Mehinto et al [9] et Mbah et al [11] qui ont trouvé respectivement que 61,69% (p = 0,42) et 58,1% (p = 0,98) de leurs patients avaient leur hernie à droite. Les différences ne sont pas statistiquement significatives. L'un des avantages de la technique de shouldice est sa faisabilité sous anesthésie locale ou locorégionale qui diminue la morbidité postopératoire. La majorité de nos patients ont été opérés sous anesthésie locale dans 48,9% et sous anesthésie locorégionale chez 40,4% et de l'anesthésie générale chez 10,7%. El Alaoui et al [12] ont trouvé que l′anesthésie locorégionale était largement utilisée chez 96,2% (p = 10-6) des patients, suivie de l′anesthésie locale chez 2,3% (p = 10-6) et de l'anesthésie générale chez 1,5% (p = 0, 001). Mais les différences sont statistiquement significatives. Ces différences pourraient être dues à la taille de notre échantillon qui est plus importante et les types de malade. Campanelli et al [13] ont trouvé une anesthésie locale chez 66,76% de leurs patients (p = 51.10-4). Cette différence peut être liée au nombre élevé de complications préopératoires (récidive et étranglement) dans notre étude, qui nécessite parfois une anesthésie locorégionale voire générale. Le fil non résorbable a été utilisé chez 93% (209) des patients et du fil résorbable chez 7% (16) pour la cure de la hernie. Nous n'avons pas trouvé de lien entre l'utilisation des fils non-resorbables et la survenue des récidives postopératoires. Les suites opératoires immédiates (0 à 8 jours) étaient simples chez 86,7% des patients, suivi de l'existence d'un hématome inguinal ou scrotal chez 8% et de la douleur aigue chez 5,3%.

Nos résultats sont inférieurs à ceux obtenus par El Alaoui et al [12] qui ont trouvé que les suites opératoires immédiates étaient simples chez 92,4% des patients, suivi d'un hématome pariétal chez 3% et de l’œdème scrotal chez 1,5%. Ailleurs, Jacquet et al [14] ont trouvé en postopératoire immédiat, que 33,6% des patients avaient une symptomatologie douloureuse postopératoire. Dans les suites opératoires immédiatesR Sani et al [15] ont trouvé que 28% des patients avaient de petits hématomes et14% de douleurs résiduelles à l′hôpital national de Niamey. Dans notre étude, on avait revu 100% de nos patients à un mois, 64% à un an, 45,8% à deux ans et 2,3% en 14 ans de recul. Nos résultats sont semblables à ceux de Millat et al [16] qui ont revu 75% de ses patients à un mois, 56% à un an et 67% à deux ans. Par contre Barrier et al [17] avaient perdu de vus seulement 15% de ses patients pour un recul de 14 ans. Dans notre série, 3,5% de nos patients avaient récidivé à un an de suivi. Notre taux est comparable à celui de Wassern et al [18] au Pakistan qui ont trouvé 1,28%, Harouna au Niger [19] 2% et Drew PJ et al [20] en Angleterre 0,99%. Le séjour post opératoire moyen a été de 5,9 jours avec un écart type de 3,6 jours. Certains auteurs [12, 19, 21] ont rapporté une durée d'hospitalisation posopératoire qui varie entre 3 et 5 jours.

## Conclusion

La technique de Shouldice est la technique de choix pour la cure de la hernie inguinale dans les pays en développement à cause du bon résultat. Elle a pour avantage de pouvoir être réalisée dans toutes les circonstances, en utilisant un matériel simple et peu coûteux, tout en assurant une réparation pariétale de qualité. Il est donc indispensable pour tout chirurgien exerçant dans les régions sous médicalisées de connaître cette technique, qui a fait la preuve de son efficacité et de sa reproductibilité. En outre l'impact à long terme des dispositifs médicaux (prothèse) utilisés dans la cure de la hernie inguinale n'est pas encore totalement élucidé.
